# Sexual Behaviors and HIV/STI Prevention Strategies Among Sexual Minority Men in Ecuador Who Use Geosocial Networking Apps

**DOI:** 10.1007/s10508-021-02093-7

**Published:** 2021-09-28

**Authors:** Carlos Hermosa-Bosano, Clara Paz, Paula Hidalgo-Andrade, Rodrigo Aguayo-Romero

**Affiliations:** 1 School of Psychology, Universidad de Las Américas, Redondel del Ciclista, Antigua Vía a Nayón, Campus UDLAPark, Quito, 170124 Ecuador; 2 Brigham and Women’s Hospital, Harvard Medical School, The Fenway Institute, Boston, MA USA

**Keywords:** Geosocial networking, Mobile apps, Gay dating apps, Men who have sex with men, HIV prevention strategies, Sexual orientation

## Abstract

Around the world, geosocial networking apps have become widely popular among sexual minority men (SMM). This research analyzed the sexual behaviors and HIV and STI prevention strategies (HIV/STI testing, HIV/STI inquiry, and HIV/STI disclosure) of an online-recruited sample of 284 SMM living in Ecuador. Sexting and oral sex were the most common sexual behaviors among SMM in the sample. Most participants had low perceptions of HIV and STI risk; 85% reported being tested for HIV and 70% for STIs. Being older predicted higher odds of being tested for either HIV or STIs at least once. Being single also predicted HIV testing. Future interventions in the country should explore apps’ utility as intervention tools to spread information about sexual health and HIV prevention strategies, such as condom use and event-driven PrEP. Apps can also facilitate connections to sexual health services, including programs for PrEP initiation and linkage to HIV treatment. They should also focus on promoting sexual harm reduction conversations among potential app-met sexual partners.

## Introduction

According to UNAIDS (2019b), in 2018, an estimated amount of 100,000 people in Latin American countries received a new HIV diagnosis. Sexual minority men (SMM), transgender women, people who engage in sex work, and people who use drugs accounted for approximately 65% of the new infections in the region (UNAIDS, 2019b). SMM are among the most vulnerable groups, with an HIV risk 22 times higher than the general population (Singh et al., 2018; UNAIDS, 2019b). In addition, SMM are at high risk of acquiring sexually transmitted infections (STIs) such as herpes, syphilis, chlamydia, and gonorrhea (Unemo et al., 2017; Yuan et al., 2019), thereby increasing the odds of subsequent HIV infections (Harney et al., 2019).

By 2019, UNAIDS data indicate that there were approximately 47,000 adults and children living with HIV in Ecuador, and that HIV prevalence was 0.4% among those between 15 and 49 years old (UNAIDS, 2019a). A 2017 report based on a sample of 748 SMM estimated an HIV prevalence of 16.1% in Quito and 11.2% in Guayaquil, the country’s most populated cities (INSPI & Corporación Kimirina, 2017). Regarding STIs, studies have found a herpes simplex type 2 (HSV-2) prevalence of 14% and syphilis prevalence estimates of 5.5% (Jacobson et al., 2014). Studies of SMM populations have found that engaging in condomless receptive anal intercourse, not being in a stable relationship, having multiple male sexual partners, being involved in sex work, being raped, and using alcohol significantly increase the likelihood of having an HIV diagnosis (Hernandez et al., 2017; Jacobson et al., 2014). Minority stress-related variables, including stigma, discrimination, and internalized homophobia, are also associated with increased HIV and STI risk among SMM (Andrinopoulos et al., 2015; Hatzenbuehler et al., 2008).

Despite the increasing popularity of geosocial networking mobile applications (GSN apps), there is limited information regarding their use and the implications for SMM’s sexual health in Latin American countries such as Ecuador. Grindr, Hornet, and SCRUFF have become global online and mobile platforms for SMM to connect, especially for those under the age of 35 (Landovitz et al., 2013; Sun et al., 2018). These platforms use global positioning technologies to allow users to meet, chat, and exchange information and photographs between them. Currently, Grindr is the most popular app among SMM in the U.S. and Europe (European Centre for Disease Prevention and Control, 2017; Goedel & Duncan, 2015). However, other non-SMM-specific apps such as Tinder, and social media platforms such as Facebook and Instagram, have also become increasingly widespread among SMM for sexual and non-sexual socialization (Macapagal et al., 2018, 2020).

Due to their popularity and potential to increase users’ social and sexual networks, there has been increased interest about the implications of app use on the spread of HIV and STIs (Sun et al., 2018; Wang et al., 2018; Zou & Fan, 2017). Studies on the matter have focused on the analysis of sexual risk behaviors (i.e., condomless anal intercourse; group sexual activity; drug use or chemsex) as well as preventive strategies among app users (e.g., HIV/STI testing, HIV/STI disclosure) (Grosskopf et al., 2014; Holloway, 2015; Phillips et al., 2016). Altogether, research has indicated that app users statistically differ to non-users in terms of: the number of sexual partners and sexual encounters; the number and type of sexual risk behaviors; and the frequency of STIs diagnoses, such as syphilis, chlamydia, and gonorrhea (Wang et al., 2018; Zou & Fan, 2017).

In a meta-analysis of 14 studies, Zou and Fan (2017) found that 46.4% of GSN app users reported having at least one instance of condomless anal sexual intercourse in the previous 3 months. Moreover, 30% of users reported being under the influence of alcohol or drugs during their last sexual encounter, thus reducing their decision-making capabilities for condom use. Regarding HIV testing, app users are more likely to get tested, probably because they perceive their sexual behaviors (e.g., condomless sex) as high risk for HIV or STI transmission (Wang et al., 2018). Conversely, HIV and STI status disclosure and inquiry are not widespread practices among users; in Zou and Fan’s (2017) study only 50% of participants reported asking for the HIV or STI status of their sexual partners.

So far, studies on GSN apps among SMM have been mostly carried out in the Global North. In Latin America, Queiroz et al. (2019) published a study based on a Facebook-recruited sample of 2250 SMM in Brazil. Participants in this study reported using mainly Tinder and Grindr, had an average of 2.8 sexual partners in the month prior to the survey and high HIV (7.1%) and STI (11.1%) prevalence. In a sample of 312 SMM and 89 transgender women in Peru, Chow et al. (2017) found that social media users had a greater number of anonymous sexual partners and high levels of gonorrhea (30%) and chlamydia (15%). However, in their sample, Facebook and Manhunt were the apps most used for meeting sex partners.

Considering the limited scientific research in Ecuador, the high prevalence of HIV and STIs among SMM in the country, and the potential GSN apps have as intervention sites for HIV and STIs prevention (Cao et al., 2017; Contesse et al., 2020; Hightow-Weidman et al., 2015; Muessig et al., 2015), this research sought to analyze the sexual behaviors as well as HIV and STI prevention strategies of a sample of 284 Ecuadorian SMM users of GSN apps. Specifically, we pursued three research objectives: (1) describe the characteristics of SMM’s sexual behaviors during their encounters with other app users, (2) describe the HIV and STI prevention strategies adopted by the men in the sample (testing, treatment engagement, HIV and STI status inquiry and disclosure), and (3) examine whether demographic and sexual behavior variables predict the adoption of HIV and STI preventive strategies (HIV/STI testing and HIV/STI inquiry).

## Method

### Participants

We conducted an exploratory, cross-sectional study with an online-recruited sample of SMM living in Ecuador. To take part in the study, all participants had to be at least 18 years old, live in Ecuador, self-identify as male, and report having used GSN apps to meet male users in the past year. We gathered a sample of 303 participants who met the inclusion criteria. However, in this article, we analyzed data from the 284 participants who reported having sexual encounters with other app users they met. Other results from this study are reported elsewhere (Hermosa-Bosano et al., 2021).

### Procedure

Data collection took place between December 2019 and February 2020 using snowball sampling via Facebook, Instagram, Twitter, and WhatsApp. To collect the sample, the research team sent the link to contacts who were part of the population and asked them to send the survey link throughout their networks. The research team also posted the study invitation on the Facebook page of Universidad de Las Américas’ Psychology Department and that of Cerebro, Emoción y Conducta (CEC) (use italics), the research group to which the authors belong. The resulting convenience sample was asked to read an informed consent agreement and complete an anonymous online survey delivered through SurveyMonkey (SurveyMonkey Inc., 2020). Participants were advised that they would not receive monetary incentives for participation, and they could stop answering the survey at any point. The corresponding author received approval for this study from the Ethics Committee of Universidad San Francisco de Quito (Ecuador) (CEISH-USFQ code: 2019-109E).

### Measures

The survey was developed in Spanish by the research team and is available upon request to the corresponding author. An initial version of the survey was reviewed by four Ecuadorian and two Colombian experts in sexual health, HIV/AIDS, and SMM populations. After incorporating their feedback into the survey, a second version was reviewed by five GSN app users who provided feedback on its clarity, difficulty, and length. Information from this phase indicated reducing the number of questions to avoid respondent fatigue.

*Sociodemographic characteristics:* Sample characteristics were assessed by 19 questions. Variables included assigned sex at birth, gender, age, nationality, country of residence, and city of residence. Additionally, we assessed education level, self-identified race/ethnicity (based on Ecuador’s last Census), religious practicing, occupation, and average income (measured in USD, Ecuador’s official currency). We also included questions about self-identified sexual orientation, gender of their sex partners from the past year, and relationship status. If participants reported being in a relationship, we asked about relationship length and cohabitation.

*Sexual behaviors:* This section focused on the sexual behaviors that participants reported having with sex partners they met on apps in the past year. In the beginning, we included the following introductory text: “In this section, we are going to ask you about your sexual behaviors with the people you have interacted with or met through applications to meet others or to date (e.g., Tinder, Grindr, etc.) in the past year. Please respond sincerely; remember that this information is anonymous.”

The first question in this section asked about the number of sex partners they met on apps in the past year using an open-question format (“In the past year, how many people from these apps have they had met sexual relationships with?”). We then asked participants about the apps they used to meet those partners (“Which of the following apps did you use to meet them?”). This multiple-choice question included the following options: Tinder, Happn, Grindr, SCRUFF, Hornet, Manhunt, Daddyhunt, Facebook, Instagram, none, and other.

Additionally, participants had to report the sexual behaviors they had engaged in with the sex partners they met on apps. To gather data on non-physical sexual behaviors, participants were asked in a select-all-that-apply format to inform whether they had engaged in written sexting (i.e., explicit sex messaging), active and passive audiovisual sexting (i.e., sending and receiving sexually explicit photos and/or videos), and live webcam sex. Similarly, to evaluate physical sexual behaviors, participants were asked whether they had met up and engaged in mutual masturbation, insertive oral sex, receptive oral sex, receptive anal sex, and/or insertive anal sex. Participants were also asked if they had used condoms when engaging in these behaviors.

In the final part of this section, we included two items to evaluate the overall perception of risk of acquiring HIV and STIs using a 5-point Likert scale that ranged from 1 = very low to 5 = very high. The question was worded as follows: “Based on your sexual behaviors with other app users, how high do you consider your risk of getting an HIV infection or contracting other STIs?” We also included a multiple-choice question to assess substance use during sexual encounters with app-met sexual partners. Options included alcohol, energy drinks, cigarettes, marijuana, cocaine, poppers, Viagra, none, and others.

*HIV and STI prevention strategies*: The final segment of the survey included questions to assess HIV and STI testing practices (“Have you ever been tested for HIV in your lifetime?”, “Have you ever been tested for STIs in your lifetime (excluding HIV)?”) and time since last testing (“When was the last time you had an HIV test?”, “When was the last time you were tested for any of the following STIs?”). We also asked whether they knew if they had a positive HIV status through a single-choice question that included the options “Yes,” “No,” “I don’t know” and “I don’t want to share this information.” Regarding STIs, we asked whether they had ever tested positive for syphilis, chlamydia, gonorrhea, and/or herpes; options also included “others,” “none,” “I don’t know” and “I don’t want to share this information.”

If participants reported a positive HIV status or previously/currently testing positive for STIs, they were asked about current/past medical treatment, whether they had disclosed their status to their sexual partners (i.e., met on apps, met outside of GSN apps), and how they disclosed (i.e., face-to-face conversations, telephone call, text/voice messaging). Participants without positive diagnoses were presented questions about pre-exposure prophylaxis (PrEP) use. All participants—regardless of their HIV status—were also asked whether they had inquired about their partners’ HIV and STIs statuses—both for partners met on apps and outside of apps—and how they asked their partners (i.e., face-to-face conversations, telephone call, text/voice messaging).

### Statistical Analyses

IBM SPSS version 25 (IBM Corp. Inc., 2017) was utilized for data analysis. Descriptive statistics were used to characterize the sample and the overall responses. Independent sample *t* tests were used to compare HIV and STI risk perceptions based on self-reported HIV status. Multiple logistic regression models were performed to predict HIV testing (0 = not tested, 1 = tested for HIV at least once), STI testing (0 = not tested, 1 = tested for STIs at least once), HIV inquiry (0 = does not ask about HIV status, 1 = asks about HIV status), and STI inquiry (0 = does not ask about previous or current STIs, 1 = asks about previous or current STIs). Sociodemographic predictors included age, education level (0 = K-12 education, 1 = higher education), race/ethnicity (0 = other, 1 = multiracial), income (0 = minimum wage or lower, 1 = higher than minimum wage), relationship status (0 = not in a relationship, 1 = in a relationship). Sexual behavior predictors included the number of sex partners, number of apps used, number of non-physical sexual behaviors, number of physical sexual behaviors, number of substances used, HIV risk perception, and STI risk perception. Log-2 transformations were carried out to correct for positive skewness. Only significant predictors will be reported.

## Results

### Sample Characteristics

Table 1 provides a complete description of SMM participants in the sample. Participants’ age ranged from 18 to 62 years old (*M* = 26.5 years, SD = 6.62). In terms of gender and sexual orientation, 99.6% (n = 283) reported being cisgender males, 78.2% self-identified as gay (*n* = 222) and 21.8% as bisexual (*n* = 62). Most participants reported living in Quito (71.8%, *n* = 204), identified mostly as multiracial (mestizo, 83.5%, *n* = 237), practiced a religion (41.9%, *n* = 119), had higher education (63.4%, *n* = 105), and reported working at the time of the study (41.8%, *n* = 119).

**Table 1:** Characteristics of participant SMM (*n* = 284)

	*n* (%)
Age (*n* = 279; *M* = 26.5, SD = 6.6)	
18–24	128 (45.9)
25–34	118 (42.9)
35–44	26 (9.3)
45–64	7 (2.5)
Sex (*n* = 284)	
Male	282 (99.3)
Female	1 (0.35)
Intersex	1 (0.35)
Gender (*n* = 284)	
Cisgender	283 (99.6)
Transgender	1 (0.4)
Sexual orientation (*n* = 284)	
Homosexual/Gay	222 (78.2)
Bisexual	62 (21.8)
Nationality (*n* = 284)	
Ecuadorian	259 (91.2)
Other	25 (8.8)
City of Residence (*n* = 284)	
Quito	204 (71.8)
Guayaquil	38 (13.4)
Other	42 (14.8)
Race/Ethnicity (*n* = 284)	
Multiracial (Mestizo)	237 (83.5)
White	25 (8.8)
Black (Afroecuadorian)	4 (1.4)
Indigenous	8 (2.8)
Montubio (Mestizo–Coastal region)	6 (2.1)
Other	2 (0.7)
Religion practice (*n* = 284)	
Yes	119 (41.9)
Highest education completed (*n* = 284)	
Primary school	1 (0.4)
High school	102 (35.9)
Technical degree or certificate	37 (13)
Undergraduate degree or certificate	105 (37)
Graduate degree or higher	39 (13.7)
Occupation (*n* = 284)	
Working	119 (41.8)
Studying	78 (27.5)
Working and studying	51 (18)
Unemployed	36 (12.7)
Monthly income (USD) (*n* = 284)	
Less than 394 USD (Minimum wage)	120 (42.3)
395 to 1182 USD	125 (44)
More than 1183 USD (More than 3 minimum wages)	39 (13.7)
Relationship status (*n* = 284)	
In a stable relationship, without cohabitation	34 (12)
In a stable relationship, with cohabitation	25 (8.8)
Currently not in a relationship	225 (79.2)
Length of relationship (*n* = 59)	
Less than 1 year	30 (50.8)
More than 1 year	29 (49.2)
Sexual partners in the last year (*n* = 284)	
Only male	246 (86.6)
Both male and female	38 (13.4)
Number of Sexual Partners (*n* = 284, Mdn = 5, IQR = 8)	
1–5	160 (56.3)
6–10	71 (25)
11–20	30 (10.6)
21–50	16 (5.6)
Apps used (*n* = 284, *M* = 2.09, SD = 1.22)	
Grindr	241 (84.9)
Tinder	128 (45.1)
Facebook	101 (35.6)
Instagram	75 (26.4)
Manhunt	16 (5.6)
SCRUFF	8 (2.8)
Hornet	8 (2.8)
Happn	2 (0.7)
Daddyhunt	1 (0.4)
Self-reported HIV Status (*n* = 241)	
Living with HIV	26 (10.8)
Living without HIV	189 (78.4)
Doesn't know	9 (3.7)
Doesn't want to share information	17 (7.1)
Self-reported previous or current STIs (*n* = 284)	
Syphilis	30 (10.6)
Gonorrhea	23 (8.1)
Herpes (HSV-2)	20 (7)
Chlamydia	8 (2.8)
Other	13 (4.6)

Most of respondents, 79.2%, were not in a relationship at the time of the study (*n* = 225); 86.6% reported having sex only with cisgender men during the last year (*n* = 246), whereas 13.4% had sex with both cisgender men and cisgender women (*n* = 38). The number of reported sex partners met on apps ranged from 1 to 100 and the median was five (*M* = 9.68, *SD* = 15.05). Most of respondents used Grindr to meet their sex partners (84.9%, *n* = 241), with Tinder being the second most used app (45.1%, *n* = 128), followed by Facebook (35.6%, *n* = 101) and Instagram (26.4%, *n* = 75). The number of apps participants used ranged from 1 to 9, and the average number was two (*M* = 2.09, *SD* = 1.22).

Regarding HIV status, 10.8% participants said they lived with HIV (*n* = 26), 78.4% reported being HIV-negative (*n* = 189), 3.7% were unaware of their HIV status (*n* = 9), and 7.1% refused to answer the question (*n* = 17). In addition, syphilis (10.6%, *n* = 30) and gonorrhea (8.1%, *n* = 23) were the most common STIs among the men in the sample.

### Sexual Behaviors

#### Non-Physical and Physical Sexual Behaviors

The mean number of non-physical behaviors was 2.2 (SD = 1.36), with four behaviors presented in the survey. Written sexting was the most common non-physical behavior (75.7%, *n* = 215), followed by passive audiovisual sexting (63.7%, *n* = 181), and active audiovisual sexting (53.2%, *n* = 151). Live webcam sex (24.6%, *n* = 70) was the least common behavior. Regarding physical sexual behaviors, participants, on average, reported having engaged in four out of the five behaviors presented in the survey (*M* = 3.99; *SD* = 1.12). Receptive oral sex was the most common behavior among respondents (85.6%, *n* = 243), followed by insertive oral sex (83.5%, *n* = 237), masturbation (81.7%, *n* = 232), insertive anal intercourse (76.4%, *n* = 217) and receptive anal intercourse (71.5%, *n* = 203).

#### Condom Use

Descriptive analyses using the total sample indicated that 67.3% of participants said they used condoms during insertive anal sex and 62% used them during receptive anal sex. Condom use was least common when masturbating (32.7%), having insertive oral sex (14.4%), and receptive oral sex (12.7%). Analyses based on self-reported HIV status indicated that condom use during oral sex, either insertive or receptive, is rare among participants living with HIV (*n* = 26) and those who said they were HIV-negative (*n* = 189). Table 2 presents disaggregated data on condom use based on self-reported HIV status.

**Table 2:** Physical sexual behaviors and condom use based on self-reported HIV Status

		Self-reported HIV Status (*n* = 241)			
		HIV + (*n* = 26)	HIV − (*n* = 189)	Does not know (*n* = 9)	Does not want to answer (*n* = 17)
		*n* (%)	*n* (%)	*n* (%)	*n* (%)
Masturbation	Without condom	22 (84.6)	128 (617.7)	6 (66.7)	8 (47.1)
	With condom	4 (15.4)	61 (32.3)	3 (33.3)	9 (52.9)
Insertive oral sex	Without condom	24 (92.3)	155 (82)	7 (77.8)	16 (94.1)
	With condom	2 (7.7)	34 (18)	2 (22.2)	1 (5.9)
Receptive oral sex	Without condom	25 (96.2)	161(85.2)	8 (88.9)	12 (70.6)
	With condom	1 (3.8)	28 (14.8)	1 (11.1)	5 (29.4)
Receptive anal sex	Without condom	10 (38.5)	66 (34.9)	2 (22.2)	4 (23.5)
	With condom	16 (61.5)	123 (65.1)	7 (77.8)	13 (76.5)
Insertive anal sex	Without condom	7 (26.9)	58 (30.7)	3 (33.3)	4 (23.5)
	With condom	19 (73.1)	131 (69.3)	6 (66.7)	13 (76.5)

#### Substance Use and Risk Perception

Over half of respondents (52.1%, *n* = 148) indicated having used at least one substance during their sexual encounters. Alcohol was the most frequently consumed substance (43.0%, *n* = 122), followed by cigarettes (19.7%, *n* = 56), poppers (16.2%, *n* = 46), marijuana (13.7%, *n* = 39), energy drinks (8.1%, *n* = 23), cocaine (4.9%, *n* = 14), Viagra (3.5%, *n* = 10), and others (1.8%, *n* = 5).

Regarding HIV risk perception, 59.8% (*n* = 113) of the 189 participants living without HIV reported their risk to be either very low (29.6%, *n* = 56) or low (30.2%, *n* = 57). An independent sample *t* test indicated no differences in the HIV risk perceptions among participants without HIV who used condoms while engaging in receptive anal sex and those who did not use condoms (*t*(187) = 0.35, *p* = 0.724). Similarly, no statistical differences were detected among participants not living with HIV who used condoms and participants who did not use condoms while having insertive anal sex (*t*(187) = 1.25, *p* = 0.213).

As for STI risk perception, 58.6% (*n* = 166) of the total number of participants (i.e., participants living with HIV and not) perceived their STIs risk to be very low (27.8%, *n* = 79) or low (30.6%, *n* = 87). Participants living with HIV perceived higher levels of STI risk (*M* = 3.00, *SD* = 1.32, *n* = 26) compared with those not living with HIV (*M* = 2.26, SD = 1.12, *n* = 189), *t*(213) = 3.03, *p* = 0.003. Independent sample *t* tests among participants living with HIV indicated no differences in the STI risk perceptions among those who used condoms and those who did not use condoms when engaging in receptive (*t*(24) = 0.90, *p* = 0.373) or insertive anal sex (*t*(24) = 0.65, *p* = 0.516). Likewise, no differences were found among participants nor living with HIV who engaged in receptive anal sex (*t*(187) = 0.43, *p* = 0.666) and insertive anal sex (*t*(187) = 1.17, *p* = 0.242) with or without condoms.

### HIV and STI Prevention Strategies

#### HIV Testing and Treatment

Most individuals (84.9%, *n* = 241) in the total sample (i.e., participants living with HIV and not) reported having tested for HIV at least once in their lifetime. Forty-three participants (15.1%) reported not ever taking an HIV test. Among participants living with HIV (*n* = 26), 61.5% (*n* = 16) reported being tested less than a year before taking the survey. In the case of participants without HIV (*n* = 189), 78.8% (*n* = 149) reported being tested less than a year before taking the survey. As for treatment, most of the participants living with HIV reported being in Highly Active Antiretroviral Therapy (HAART, 80.8%, *n* = 21). Only 5.3% (*n* = 10) of the participants without HIV reported using PrEP.

#### HIV Inquiry and Disclosure Practices

Almost half of the total sample (i.e., participants living with HIV and not) asked their sexual partners about their HIV status (47.9%, *n* = 136). Ten of the 26 participants living with HIV (38.5%) asked their partners about their HIV status. Most of them asked their partners’ HIV status during face-to-face encounters (80.0%, *n* = 8); 1 participant reported using text or voice messages (10.0%) and another participant reported doing it through a phone call (10.0%). Among participants without HIV (*n* = 189), 53.4% (*n* = 101) reported asking about their partners’ HIV status. The majority (87.1%) reported doing it during face-to-face encounters (*n* = 88), 10.9% via text or voice messages (*n* = 11), 2% reported doing it in other ways (*n* = 2).

Regarding HIV status disclosure practices, nine out of the 26 participants living with HIV (34.6%) shared their status with their app-met sexual partners; all of them indicated using face-to-face conversations.

#### STI Testing and Treatment

Almost 70% (*n* = 197) of the participants in the total sample (i.e., participants living with HIV and not) reported having been tested for STIs during their lifetime. Sixty-four participants (22.5%) from the total sample reported having had at least one STI diagnosis in their lifetime. Of those, 92.2% (*n* = 59) received medical treatment. Analyses based on HIV status indicate that most participants living with HIV (92.3%, *n* = 24) reported having tested for STIs at least once. Most of them reported that their last STI test was taken less than a year prior to the survey (88.5%, *n* = 23). Fourteen out of the 26 participants (53.8%, *n* = 14) living with HIV reported having been treated for STIs. In the case of participants without HIV (*n* = 189), 77.8% (*n* = 147) reported having had a STI test at least once in their lifetime, with the majority reporting having been tested in the past year (78.9%, *n* = 116). Forty of the 189 participants (21.2%) indicated having had an STI and getting treated for it.

#### STI Inquiry and Disclosure Practices

Only 41.5% (*n* = 118) of participants in the total sample reported asking their sex partners about previous or current STI diagnoses. Of those who inquired about STIs, 84.7% (*n* = 100) did it during a face-to-face conversation, 13.6% with a text or voice message (*n* = 16), 0.8% during a phone call (*n* = 1), and 0.8% in another way (*n* = 1). Of the people who reported a previous or current STI (*n* = 64), only 31.3% (*n* = 20) reported telling their sex partners about their diagnosis. Most of the participants who disclosed their STI diagnoses to their partners did it during a face-to-face conversation (90.0%, *n* = 18), and only 10.0% (*n* = 2) did it with a text or voice message.

### Prediction Models for HIV and STI Prevention Strategies

Logistic regression analyses indicated a significant model when predicting HIV testing, *χ*^2^(12, N = 284) = 35.88, *p* = 0.001. Age (*B* = 2.301; OR = 9.987; *p* = 0.006) and relationship status (*B* = − 0.883; OR = 0.414; *p* = 0.05) were the only significant predictors of HIV testing. When predicting STI testing, we also found a significant model, *χ*^2^(12, *N* = 284) = 38.11, *p* < 0.001. Age was the only significant predictor of STI testing (*B* = 2.286; *OR* = 9.835; *p* < 0.001). In both cases, older people had higher odds of being tested for either HIV or STIs at least once. Those not in a relationship had a higher likelihood of being tested for HIV. Table 3 presents the logistic regression coefficients using HIV testing and STI testing as outcome variables. Analyses predicting HIV and STI inquiry practices did not yield statistically significant models based on the included descriptive and sexual behaviors variables.

**Table 3:** Logistic regression models predicting HIV and STI testing

	HIV testing					STI testing				
	*B*	Wald *χ*^2^	OR	95% CI		*B*	Wald *χ*^2^	OR	95% C.I	
				Lower	Upper				Lower	Upper
Constant	− 10.15	7.308				− 11.11	16.47			
Demographic variables										
Age (log-2)	2.30**	7.51	9.99	1.92	51.7	2.29**	14.83	9.84	3.07	31.4
Education level	.728	2.87	2.07	.892	4.81	.444	1.93	1.56	.833	2.92
Race/ethnicity	− .442	.626	.643	.215	1.92	.278	.486	1.32	.604	2.88
Religious practice	− .081	.047	.922	.445	1.91	− .231	.425	.794	.451	1.40
Relationship status	− .883*	3.806	.414	.170	1.04	-.208	.572	.812	.395	1.67
Sexual behaviors										
Number of sex partners (log-2)	.327	2.97	1.39	.956	2.01	.013	.009	1.01	.781	1.31
Number of used apps (log-2)	.696	3.04	2.01	.917	4.39	.364	1.47	1.44	.799	2.59
Number of non-physical sexual behaviors	− .196	1.66	.822	.610	1.11	.029	.059	1.02	.817	1.29
Number of physical sexual behaviors	.137	.699	1.15	.832	1.58	.023	.029	1.02	.789	1.33
Number of substances (log-2)	.134	.314	1.14	.716	1.82	.213	1.37	1.24	.866	1.77
HIV risk perception	.464	2.42	1.59	.887	2.85	.130	.317	1.14	.724	1.79
STI risk perception	− .400	1.92	.670	.381	1.18	− .223	.979	.800	.515	1.24
Chi-Square	35.88	d*f* = 12				38.1	d*f* = 12			
Nagelkerke *R*^2^	.214					.183				
Cox & Snell *R*^2^	.123					.130				

**p < .01, *p < .05

## Discussion

This study sought to identify sexual behaviors, HIV and STI prevention strategies in an online-recruited sample of SMM in Ecuador who use GSN apps. It also examined whether demographic and sexual behavior variables predicted the adoption of HIV and STI prevention strategies. Data from our study indicated that Grindr, an SMM-specific dating app, was the most frequently used platform for meeting sex partners, followed by other non-SMM targeted apps, such as Tinder, and social media platforms, like Facebook and Instagram. As noted in previous studies, Grindr has become widely popular among SMM because it allows users to instantly engage in conversations that often lead to the establishment of new friendships, romantic relationships, and sexual encounters (Goedel & Duncan, 2015; Holloway, et al., 2014a, 2014b; Rice et al., 2012; Zervoulis et al., 2020). The relative anonymity it provides its users—allowing them to express their sexual interests through the privacy of their devices—might be relevant in Ecuador’s context. International polls reveal that, despite there has been an increase in the levels of acceptance toward homosexuality over time, Ecuador is still far from being an accepting country for sexual and gender minorities (Pew Research Center, 2013; Tummino & Bintrim, 2016). The social climate in the country may create the need for some SMM to find safe spaces where they can easily meet other men. Thus, using SMM-specific apps can be a way to develop new social connections, without a high risk of visibility. These apps may help create connections with the gay community and a social support system to help navigate the intricacies of living in a heteronormative, stigmatizing context, especially for those who are closeted (Zervoulis et al., 2020).

The analyses of sexual behaviors indicated that written sexting, followed by passive and active audiovisual sexting, were the most frequent forms of non-physical sexual interactions among the men in the sample. Past studies have identified that people engage in sexting behaviors to create intimacy and bonds with partners, gain money, flirt, gain romantic attention, initiate sexual activity, and arouse potential partners (Cooper et al., 2016; Drouin et al., 2013). It is possible that our participants had similar motivations; however, it is also feasible that SMM perceive sexting as a preventive strategy where they can engage in sexual interactions without being vulnerable to HIV and STIs. Furthermore, it is possible that people adopt these behaviors because they do not perceive any potential risks associated with them. Previous studies have highlighted the risks implied in sexting practices which include the possibility of being rejected or humiliated, having intimate information sent to others, interacting with people using fake profiles, cyberbullying, harassment, extortion, and others (Dir & Cyders, 2015; Drouin et al., 2017). Future research should examine the motivations behind these behaviors, whether users perceive any psychosocial benefits and risks, as well as potential differences regarding risk and preventive HIV and STI behaviors based on motivations, apps used during sexting (e.g., SMM-specific app users vs. non-SMM-specific app users) and sexting patterns (e.g., active sexters vs. passive sexters).

Regarding physical sexual behaviors, results indicate that both receptive and insertive oral sex were the most common practices, followed by mutual masturbation and, to a lesser degree, insertive and receptive anal sex. This finding, along with the data showing low perceptions of HIV and STI risk and low rates of condom use during oral sex, makes us believe that people do not conceive oral sex as a potential driver of HIV and STI transmission. Studies have shown that oral sex is an effective practice when it comes to preventing HIV; however, it is associated with the perpetuation of STIs such as gonorrhea, syphilis, and chlamydia (Glynn et al., 2017). This information is relevant considering reports of high prevalence of HIV and STI co-infections among SMM in cities like Quito (Jacobson et al., 2014). More specifically, multiple studies have determined that having an STI increases the risk of HIV infection (Hayes et al., 2010; Pathela et al., 2013; Peterman et al., 2014). Our results were no exception in this regard, since we found a significant number of participants who had tested positive for these conditions. In our study, 10.8% of participants reported living with HIV, whereas 31.8% reported testing positive for at least one STI during their lifetime, with syphilis and gonorrhea being the most common diagnoses.

Data concerning HIV and STI testing revealed that, despite most SMM reported being tested 3 months prior to the survey, a significant amount of SMM had never been tested, especially for STIs. Regression models revealed that age significantly predicted testing, indicating that older participants had higher odds of being tested for either HIV or STIs at least once. As previous research has noted, it is possible that younger SMM do not get tested due to multilevel factors that act as barriers. These might include concerns about confidentiality, testing costs, fear of testing outcomes, partner and social rejection, healthcare provider stigma, HIV-related stigma, and gay-related stigma, among others (Lopez-Quintero et al., 2005; Pharr et al., 2015).

Regarding treatment, this study indicated that most of individuals living with HIV, and those who reported having had an STI, were being treated or had previously received treatment. In terms of prevention, only 8.5% of participants reported using PrEP, probably due to limited availability to this form of prevention, lack of access, or even lack of awareness about these options. PrEP has only been available in Quito and Guayaquil since the middle of 2019 and is just now gaining uptake among SMM in the country. Our research also revealed that roughly half of participants engaged in conversations about HIV and STI status. Discussions about HIV and STI status should become a regular part of the sexual negotiations that take place prior to sexual encounters and could become one of the many prevention strategies SMM in Ecuador could use. Moreover, open dialogue about HIV and STIs is needed to combat stigma surrounding these health conditions. GSN app developers can also be involved in this process by facilitating profile options where users can disclose their HIV/STI status and testing history. Some apps, such as Grindr, have started to provide those options in addition to providing links to obtain more sexual health information.

Finally, we found that approximately 30% of participants with self-reported HIV positive and STI status disclosed their conditions to their partners. Disclosure of one’s own status is a personal decision, and some app users may not feel comfortable sharing it with others to avoid rejection and discrimination, especially if their partners are people who they just met (Wang et al., 2018) and who can potentially tell other people about their HIV status. Knowing that approximately only one out of three SMM in Ecuador disclose their HIV/STI status reflects how HIV/STIs are still highly stigmatized. The onus should not be on those living with HIV/STIs to disclose their diagnoses but rather on everyone engaging in any physical sexual behavior that carries HIV/STI vulnerability.

### Limitations

There were some limitations in our study. First, we used an online survey that was distributed through social media using snowball sampling methods. These procedures led to the underrepresentation of certain groups with sociodemographic characteristics and localities (e.g., Black, Indigenous SMM) and overrepresentation of others (e.g., multiracial, higher income SMM from Quito and Guayaquil) (Burrell et al., 2012; Sullivan et al., 2011). Second, despite creating an anonymous survey, it is possible that some participants felt uncomfortable reporting details of their sex life, even altering important information due to social desirability effects.

### Implications

Despite limitations, we believe our study has several practical implications. Previous studies have highlighted that GSN apps can become intervention tools to: increase awareness of HIV, STIs, and PrEP; distribute HIV self-test kits; highlight the importance of consistent condom use; and link SMM with HIV testing, prevention, and treatment centers at nearby localities (Cao et al., 2017; Contesse et al., 2020; Holloway, et al., 2014a, 2014b; Jenkins Hall et al., 2017; Su et al., 2015). To our knowledge, there are no current initiatives in Ecuador to utilize apps’ intervention potentials. Based on its widespread use among the SMM in our sample, using Grindr to deliver interventions could be useful to provide users tools to make informed decisions about which HIV and STI prevention strategies are best suited for them. Grindr has successfully implemented HIV and STI prevention interventions in other countries, such as in Bulgaria, where they provided HIV self-test kits to app users (Single Step, 2019) or in the U.S. where they have promoted syphilis testing (Su et al., 2015). Implementation science studies are needed to assess the acceptability and feasibility of HIV and STI prevention interventions using GSN apps in the Ecuadorian sociocultural context.

Whether through apps or other implementation methods, HIV prevention interventions in Ecuador should focus on spreading information and access to comprehensive HIV prevention strategies that include condom use, PrEP, treatment as prevention, as well as information about the potential psychosocial benefits and risks of both non-physical and physical sexual behaviors. These interventions should explicitly present non-physical sexual behaviors as an alternative that carries no HIV/STI risk because there is no transmission of bodily fluids.

Increasing awareness and access to PrEP is a timely intervention in Ecuador since the dissemination of PrEP is still in its early stages across the country. Moreover, event-driven PrEP (ED-PrEP), also known as the 2 + 1 + 1 dosing, is an attractive delivery method that should be considered in Ecuador given its history of delays and shortages of HAART (Basantes, 2020). This method entails the use of a double dose of PrEP between 2 and 24 h in advance of sex; a third pill 24 h after the first intake, and a fourth pill 24 h after the second intake (World Health Organization, 2019). This strategy would allow Ecuadorian SMM to make their monthly supply last longer in anticipation of shortages of supply and will ensure that SMM have PrEP available when they need it (Basantes, 2020).

Lastly, structural interventions should address the issue of constant shortages of HAART supply that can undermine the efficacy of treatment for people living with HIV and the efficacy of treatment as prevention (Centers for Disease Control & Prevention, 2018). Educational campaigns that encourage HIV/STI inquiry and disclosure are also needed to increase the number of people engaging in those behaviors. Those educational efforts would highly benefit from incorporating the prevention message that Undetectable = Untransmittable (U = U) to inform SMM in Ecuador that people with undetectable viral loads do not transmit HIV. Structural interventions also need to address sexual orientation, HIV and STI stigma in tandem with educational campaigns to enact changes in attitudes among the Ecuadorian population.

Regarding future studies, we believe that it would be useful to develop research to compare app users versus non-app users, as well as younger and older SMM. Studies should consider using qualitative methods to further analyze the experiences of app users and identify context-related aspects that help explain the sexual behaviors of SMM as well as the individual, interpersonal and structural barriers and facilitators associated with HIV and STI prevention. Finally, we believe it would be useful for researchers to analyze app use among other vulnerable groups such as transgender and non-binary populations.

### Conclusion

To our knowledge, this is the first study in Ecuador to analyze sexual behaviors as well as HIV and STI prevention strategies in a sample of SMM who use their mobile devices as means to meet sexual partners. We found the existence of behaviors that may increase HIV and STIs vulnerabilities, as well as other potential psychosocial risks. Moreover, this study yielded valuable data to direct the development of future interventions to facilitate decision-making processes among vulnerable populations.

## Data Availability

The data that support the findings of this study are available from the corresponding author upon reasonable request.
